# Prediction of ovulation: new insight into an old challenge

**DOI:** 10.1038/s41598-023-47241-2

**Published:** 2023-11-15

**Authors:** Ettie Maman, Eli Y. Adashi, Micha Baum, Ariel Hourvitz

**Affiliations:** 1 Sheba Medical Center In Vitro Fertilization Unit, Department of Obstetrics and Gynecology. Sackler School of Medicine, Tel Aviv University, Tel Aviv-Yafo, Israel; 2 Herzliya Medical Center, In Vitro Fertilization Unit, Herzliya, Israel; 3 Departments of Medical Science and Obstetrics and Gynecology, the Warren Alpert Medical School, Brown University, Providence, RI 02906 USA; 4 Shamir Medical Center In Vitro Fertilization Unit, Department of Obstetrics and Gynecology. Sackler School of Medicine, Tel Aviv University, Tel Aviv-Yafo, Israel

**Keywords:** Reproductive biology, Endocrinology

## Abstract

Ultrasound monitoring and hormonal blood testing are considered by many as an accurate method to predict ovulation time. However, uniform and validated algorithms for predicting ovulation have yet to be defined. Daily hormonal tests and transvaginal ultrasounds were recorded to develop an algorithm for ovulation prediction. The rupture of the leading ovarian follicle was a marker for ovulation day. The model was validated retrospectively on natural cycles frozen embryo transfer cycles with documented ovulation. Circulating levels of LH or its relative variation failed, by themselves, to reliably predict ovulation. Any decrease in estrogen was 100% associated with ovulation emergence the same day or the next day. Progesterone levels > 2 nmol/L had low specificity to predict ovulation the next day (62.7%), yet its sensitivity was high (91.5%). A model for ovulation prediction, combining the three hormone levels and ultrasound was created with an accuracy of 95% to 100% depending on the combination of the hormone levels. Model validation showed correct ovulation prediction in 97% of these cycles. We present an accurate ovulation prediction algorithm. The algorithm is simple and user-friendly so both reproductive endocrinologists and general practitioners can use it to benefit their patients.

## Introduction

Knowing the ovulation time is of great importance for both women and medical personnel. The fertility time frame is narrow and highly variable even among women who regard their menstrual cycles as regular^1,2^. Identifying the time of ovulation can help women in family planning both for achieving pregnancy and preventing it. Natural family planning can be very valuable in undeveloped countries where the population cannot afford expensive hormonal or surgical contraceptive methods however only few women correctly estimate their ovulation day^3^. Moreover, it can be helpful in cases where contraceptives cannot be used for medical reasons. Fertility physicians often use ovulation timing when timing insemination^4^ or transfer of frozen embryos^5^ in the natural cycle considered by many physicians as the preferred method^6^. The precise timing of ovulation is critical to the success of treatment and adjustment of the embryos to the endometrial developmental stage^5^.

Due to the great importance of determining the time of ovulation and the need for available and accessible natural family planning method^7^ researchers have tried to define a reliable accessible method for determining the time of ovulation^8–10^. Other researchers tried to develop available techniques or devices providing fertility window for their users^11–13^. The primary flaw in these works is the lack of consistency in defining the reference point known as ovulation, and after many years of research, the most accurate non-invasive method to determine ovulation still hasn't been discovered.

The major hormones in the ovulation process, LH (Luteinizing Hormone) estrogen, and progesterone have been widely studied. The studies mainly characterized the levels of hormones throughout the menstrual cycle and around the ovulation period^8,14^. Few of the studies have attempted to test the predictive value of the hormones mentioned to predict ovulation time^9,10,15^. These studies were done on a few patients^10^ or patients who had fertility problems^15^. In general, the hormonal tests, together with the ultrasound, are the tools available to the clinician when deciding on the time of ovulation. In this study, we decided to concentrate on the major indicators of ovulation, specifically the blood levels of LH, Estrogen, and Progesterone regarding leading follicle rupture observed by vaginal ultrasound. We present an accurate and user-friendly method for calculating ovulation time based on these parameters.

## Results

One hundred and eighteen cycles were examined in 37 participants who met the inclusion criteria and were included in the data processing. Table 1 presents the participants characteristics. The mean age of the volunteers was 33.0 ± 0.4 (mean ± SEM) and the mean BMI was 22.0 ± 0.3 (mean ± SEM). The cycle characteristics and the length of the follicular and the luteal phases are shown.

**Table 1:** Participants characteristics.

Participants/cycles	37/118
Age mean (years) ± SEM (range)	33.0 ± 0.4 (22.0–41.5)
Mean BMI ± SEM (range)	22.0 ± 0.3 (17.4–36.6)
Mean cycle length (days) ± SEM (range)	27.6 ± 0.3 (17.0–38.0)
Mean follicular phase length (days) ± SEM (range)	14.4 ± 0.2 (9–23)
Mean luteal phase length (days) ± SEM (range)	13.5 ± 0.2 (9–21)

**Figure 1:**
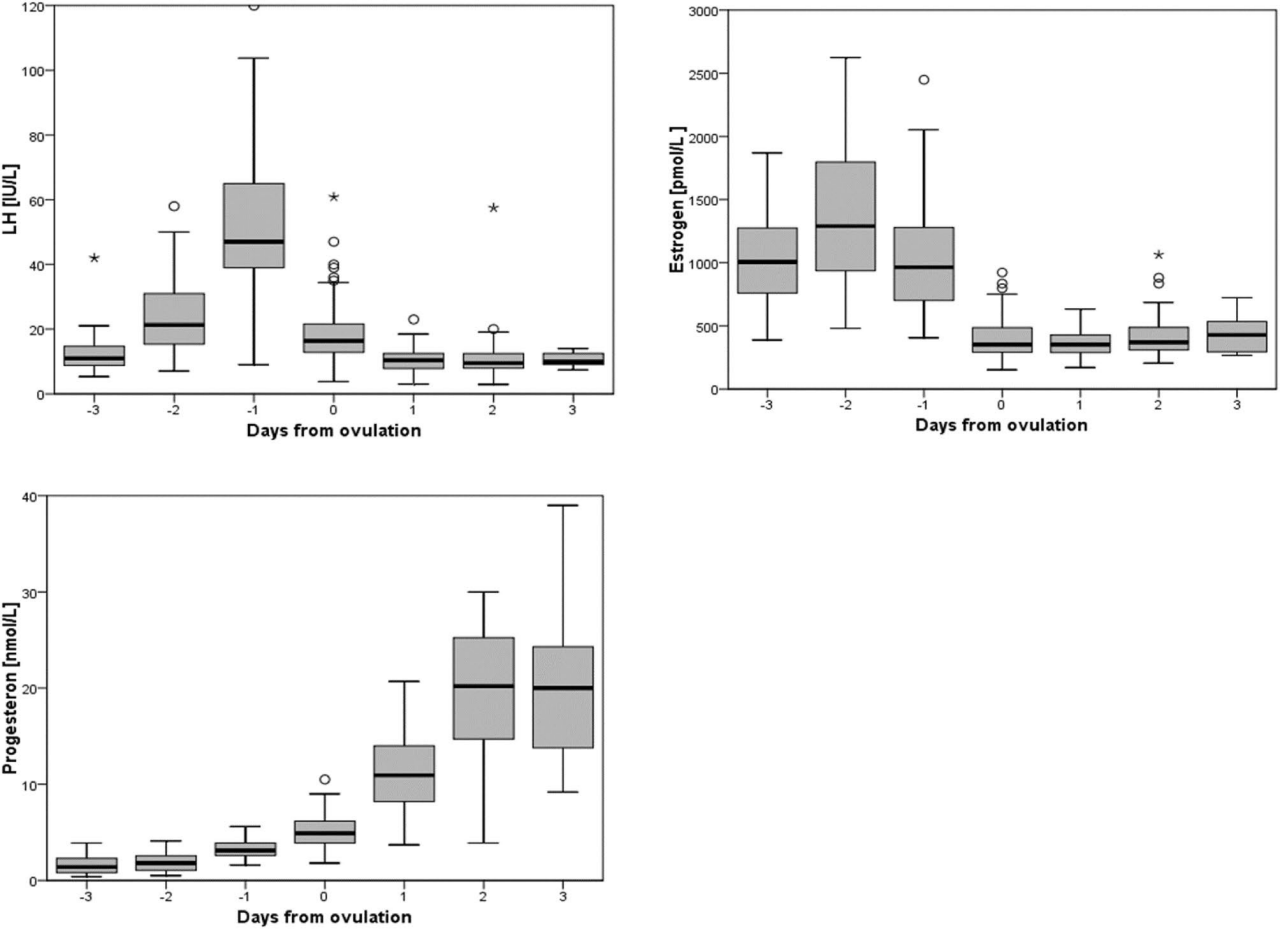
Box-plot distributions of LH, Estrogen, and Progesterone according to test day. The distribution by day of each hormone was presented using a box plot, representing five numbers summary the minimum, the first second & third quartile, and the maximum. Outliers marked by circles are either 1.5 × IQR or more above the third quartile or 1.5 × IQR or more below the first quartile. Extreme values (*) are either 3 × IQR or more above the third quartile or 3 × IQR or more below the first quartile.

Table 2 shows the overall data collected during the follow-up including estrogen, progesterone, and LH blood levels according to the follow-up days. Table 3 summarizes the relative changes in the hormone levels and Fig. 1 shows the box plot distribution of each hormone level according to the test day.

**Table 2:** Results of hormone and ultrasound follow-up.

	Day (− 3)	Day (− 2)	Day (− 1)	Day (0)	Day (+ 1)	Day (+ 2)	Day (+ 3)
n	54	64	118	118	55	42	8
LH (IU/L)							
Mean ± SEM	12.1 ± 0.7	24.2 ± 1.5	51.9 ± 1.9	18.0 ± 0.8	10.5 ± 0.5	11.3 ± 1.2	10.5 ± 0.8
(Min–max)	(5.3–21.0)	(7.0–58.0)	(89.0–120.0)	(73.8–60.8)	(3.0–23.0)	(2.9–57.5)	(7.4–14.0)
Estrogen (pmol/L)							
Mean ± SEM	1034.7 ± 49.2	1378.2 ± 66.0	1013.3 ± 35.7	393.2 ± 14.1	357.7 ± 14.7	420.5 ± 27.0	389.0 ± 68.7
(Min–max)	(388.0–1870.0)	(481.0–2625.0)	(406.0–2499.0)	(152.0–923.0)	(170.0–632.0)	(205.0–1063.0)	(267.0–723.0)
Progesterone (nmol/L)							
Mean ± SEM	1.5 ± 0.1	1.9 ± 0.10	3.2 ± 0.9	5.1 ± 0.1	11.2 ± 0.5	19.7 ± 1.0	20.2 ± 3.0
(Min–max)	(0.4–3.9)	(0.5–4.10)	(1.6–5.60)	(1.8–10.50)	(3.7–20.7)	(3.9–30.00)	(9.2–39.0)
Endometrial thickness (mm)							
Mean ± SEM	9.0 ± 0.2	9.5 ± 0.2	10.1 ± 0.2	10.1 ± 0.2	9.5 ± 0.2	9.3 ± 0.2	10.2 ± 0.8
(Min–max)	(6.5–14.0)	(2.7–14.0)	(4.3–25.0)	(6.0–19.0)	(6.0–14.0)	(6.0–13.0)	(7.0–13.0)

**Table 3:** Relative change in hormone levels between different test days.

	One day gap			Two days gap	
	D(− 3) to D(− 2)	D(− 2) to D(− 1)	D(− 1) to D(0)	D(− 3) to D(− 1)	D(− 2) to D(0)
Cases	21	63	118	54	63
Change of LH (%) mean ± SEM	(+) 133% ± 28%	(+) 183% ± 20%	(−) 59% ± 40%	(+) 395% ± 26%	(+) 4% ± 9%
Change of estrogen (%) mean ± SEM	(+) 51% ± 7%	(−) 21% ± 3%	(−) 58% ± 2%	(+)12% ± 5%	(−) 66% ± 3%
Change of progesterone (%) mean ± SEM	(+) 35% ± 12%	(+) 111% ± 12%	(+) 62% ± 5%	(+) 170% ± 20%	(+) 257% ± 29%

The peak of LH level (51.9 ± 1.9 IU/l, mean ± SEM) is measured as expected on the day before ovulation (D − 1). However, in 7 cases (5.9%) we observed the LH peak two days before ovulation (D − 2). The peak increase of LH was 183% ± 20% (2.83-fold) between D(− 2) to D(− 1). The total increase of LH between D(− 3) to D(− 1) was 395% ± 26%.

Regarding estrogen levels, we found that estrogen levels rise to peak two days before ovulation (D − 2) to an average peak of 1378 ± 66.0 pmol/l and then gradually decrease (Table 2). Initially, the level drops by an average of 21 ± 3% from D(-2) to D(-1) followed by a sharp decrease of 58 ± 2% from D(-1) to D(0) to an average of 393 pmol/l on the day of ovulation D(0) (Table 3).

As expected, a decrease in estrogen was recorded when the follicle disappeared. We found that if a drop in estrogen appeared, in 100% of the cases, the follicle disappeared the next day. However, in 19% of the cases, the drop in estrogen was observed only on the day the follicle rupture.

A sharp decrease of more than 50% in estrogen levels was observed between days D(− 2) and D0 in 85% of the cases.

Progesterone rises to 3.2 ± 0.9 nmol/L on the day before ovulation and increases to 5.1 ± 0.1 nmol/L on the day of ovulation D(0). The increase in progesterone levels starts as early as D)− 2). We saw a small change of 35 ± 12% relative to D(− 3). This increase as expected continues but the relative changes were not consistent.

The changes in endometrial thickness during the different test days are also shown in Table 2. It can be seen that as expected the endometrium thickens towards ovulation day with an average of 10.1 ± 2.4 mm on D(− 1) and the day of ovulation. Mean endometrial thickness decreased by 0.6 mm the day after to 9.5 ± 0.2 mm.

**Figure 2:**
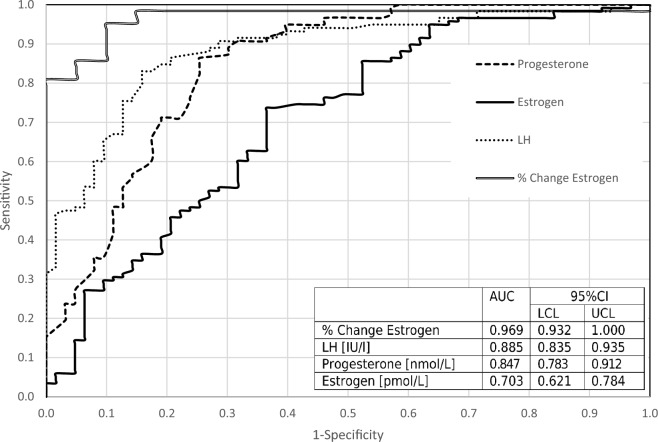
Prediction of D(− 1). ROC curves and AUC of LH estradiol and progesterone absolute levels and the percent change of estrogen level between D(− 2) and estrogen level at D(− 1). The AUC with 95% CI for each hormone is presented. The percent of change of estrogen was calculated as $Change Estrogen=\frac{{Estrogen}_{{Day}_{t}}-{Estrogen}_{{Day}_{t-1}}}{{Estrogen}_{{Day}_{t-1}}}$, *LCL* low confidence level, *UCL* upper confidence level.

To test the ability of absolute hormone values or the relative changes in hormone levels from day to day to predict ovulation time, ROC analysis was performed and AUC values were calculated. Figure 2 shows the D( − 1) discriminative capability of LH, progesterone, and estrogen absolute levels, and the percent of change in estrogen. The most reliable predictor was the percent of change in estrogen (i.e. decrease) levels from D(− 2) to D(− 1) as we can see in Fig. 2, with the AUC result of 0.969. The absolute level of LH had the highest predictability for D(− 1) of the three hormones absolute levels tested, AUCs for LH and progesterone were 0.885 and 0.847, respectively. The relative changes of LH and progesterone between D(− 2) and D(− 1) were found to have lower predictive value (Data not shown). Any drop of estrogen predicted ovulation the next day with 100% specificity and a sensitivity of 81.2% (Table 3). If estrogen decline is documented and the follicle is still present in an ultrasound test, 100% certainty can be said to be D(− 1) and ovulation will occur the next day.

For diagnosis of D(− 1) or D(0) using the change in estrogen level, we tested the significance of a sharp decrease in estrogen by 50% or more compared to the day before. We found that a drop of 50% or more in estrogen levels does have 96.4% PPV to define the ovulation day.

Following the ROC analysis, we carefully examined the coordinates of the ROC Curve tables, to find cutoff values with optimal predictive capacity in terms of specificity and sensitivity to predict ovulation time. These cutoff values can serve the clinician during the decision-making process for determining ovulation. Two LH cutoff values were selected (Table 4). LH levels equal to or more than 35IU/L had 83.0% sensitivity to ovulation detection the next day (82.2% specificity and 82.3% PPV). A threshold of ≥ 60 IU/L resulted in 100% specificity and PPV, yet a very low sensitivity of 29.7%. As noted earlier, the ROC analysis for relative change at the LH levels produced unsatisfactory results (AUC = 0.586). Further analysis of two relative changes in LH levels between D(− 2) to D(− 1) selected (relative changes of ≥ 100% and ≥ 200%) demonstrate the meager contribution of the percent change in the LH level to predict ovulation time (Table 4). We conclude that no advantage for these parameters is found over the absolute indices.

**Table 4:** Predictive values for ovulation of LH estrogen, and progesterone cutoffs.

	Sensitivity	Specificity	PPV	NPV	Accuracy
Pre-ovulatory prediction—D(− 1) (analysis vs. D(− 2))					
Any decrease in Estrogen levels	81.2%	**100%**	100%	84.2%	90.6%
Value, 95%CI	69.5–89.9%	94–100%		76.2–89.9%	84.2–95.0%
LH cutoff level					
LH ≥ 35 IU/L	83.0%	82.2%	82.3%	82.9	82.6%
Value, 95% CI	75.0–89.3%	74.1–88.6%	75.8–87.4%	76.3–87.9%	77.2–87.2%
LH ≥ 60 IU/L	29.7.0%	100%	100%	58.7%	64.8%
Value, 95% CI	21.6–38.8%			55.8–61.5%	58.3–70.9%
LH increase					
≥ 100%	65.0%	52.4%	57.7%	60.0%	58.7%
Value, 95% CI	52.0–76.7%	39.4–65.1%	50.0–65.2	49.9–69.3%	49.6–67.4%
≥ 200%	39.7%	85.7%	73.5%	58.7%	62.7%
Value, 95% CI	27.6–52.8%	74.6–93.2%	58.5–84.5%	53.1–64.0	53.6–71.5%
Progesterone					
> 2 nmol/L	91.5%	62.7%	71.0%	88.1%	77.1%
Value, 95% CI	85.0–95.9%	53.3–71.4%	65.9–75.7%	80.1–93.1%	71.2–82.3%
Post-ovulatory prediction—D(0) vs. D(− 1)					
Progesterone					
≤ 5 nmol/L	55.0%	96.6%	94.2%	68.3%	75.8%
Value, 95% CI	45.7–64.2%	91.5–99.0%	85.9–97.7%	63.7–72.5%	69.9–81.2%
Post-ovulatory prediction—D(+ 1) and D(+ 2) analysis vs D(0)					
Progesterone					
≥ 9 nmol/L	91.5%	62.7%	71.0%	88.1%	77.15%
Value, 95% CI	85.0–95.6%	53.3–71.4%	65.9–75.7%	80.1–93.1%	71.2–82.3%

Significant values are in bold.

The ROC analysis demonstrated that absolute progesterone values had less predictive ability than the estrogen relative change or absolute LH value (Fig. 2). Progesterone level of 2 nmol/L was the cutoff with the best sensitivity × specificity index, with 91.5% sensitivity to predict ovulation the next day, however, the specificity was low and insufficient at 62.7% (Table 4).

**Figure 3:**
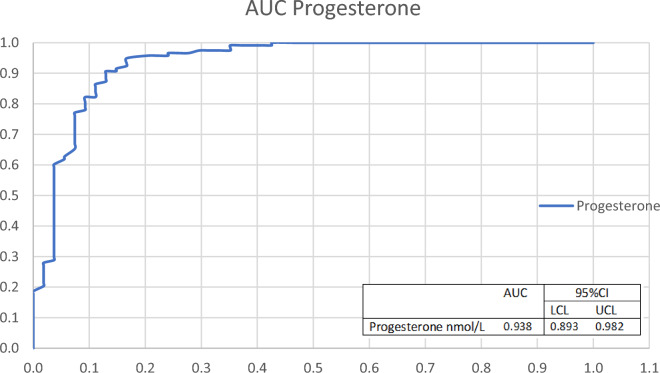
Prediction of D(0). ROC curves and AUC with 95% confidence interval of progesterone absolute levels. *LCL* low confidence level, *UCL* upper confidence level.

Regarding the post-ovulatory period, ROC analysis of hormones' absolute levels showed low AUC with no contribution of LH or estrogen levels. However, progesterone absolute levels were found to be satisfactory for D(0) prediction versus day(+ 1) in the post-ovulatory period (Fig. 3). We found that if progesterone levels were up to 5 nmol/L, the PPV for D(0) was 94.3% with a specificity of 99.6%, yet low and low sensitivity of 55.9% (Table 4). If progesterone is above 9 nmol/L, we could predict that we are at D(+ 1) or D (+ 2) with a sensitivity of 75.4% and a specificity of 99.2% (Table 4).

**Figure 4:**
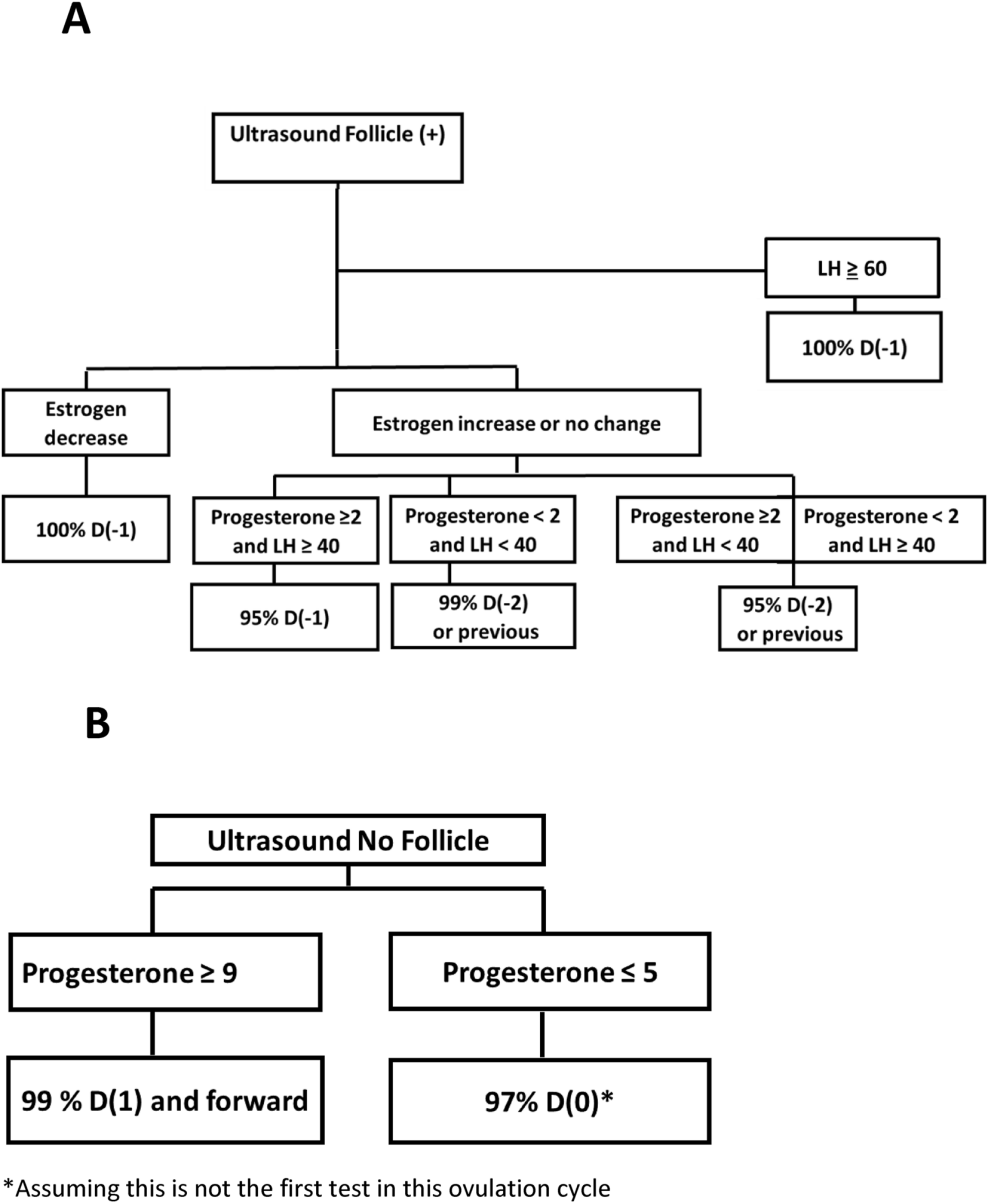
Decision process to determine ovulation time based on hormone and ultrasound tests.

Since no single individual hormone was found to be satisfactory for predicting ovulation both in terms of specificity and sensitivity, we decided to establish an algorithm that uses the results obtained for each hormone. In Fig. 4, an algorithm analysis is presented, which, is practical for clinical use and reaches the best possible prediction of ovulation.

The basic parameter is the existence or absence of the follicle. If a follicle is found on the ultrasound, then the clinician has to identify whether ovulation is going to occur the next day or two days later (Fig. 4A). With estrogen, LH, and progesterone values, the right day can be set at 95–100%. In the next step of the algorithm, the change in estrogen (i.e., the decrease) was chosen due to the combination of maximum specificity and sensitivity to predict ovulation the following day (Fig. 2). In all cases where the follicle is still present but there is a decrease in estrogen compared to a previous examination, in 100% of the cases we followed, ovulation was observed the next day. In cases where the follicle is still present and estrogen is unchanged or increased there are several possibilities and LH and progesterone values should be used. If the progesterone level is above ≥ 2 nmol/L and LH ≥ 40IU, in 95% of the cases it can be stated that it is Day (− 1). In all other cases, Day (− 2) can be determined in 95–99% of the cases (Fig. 4A).

Regarding the post-ovulatory period, in the absence of the follicle in the ultrasound and provided that follicle development was observed earlier, the only tool available to the clinician is the level of progesterone. We found that when it is 5 nmol/L or less, in 96.6% of cases it will be the day of ovulation D(0) (Figs. 3, 4B). When progesterone is above 9 nmol/L, in 99% of cases it will be the day after ovulation D(+ 1) and forward.

To validate our model, NC FET cycles were used. Ninety-five cycles met the criteria defined for validating the model's ability to predict D(− 1). In 93 cases a correct prediction was found by the model (97.9%). In 7 cases (9.5%) of those, another test set was needed the day after.

In testing the predictive ability of the model on D(0), 113 cases met the inclusion criteria. As mentioned earlier, during the post ovulatory period the model failed to demonstrate predictive values of progesterone between 5 and 9 nmol/L. Of the 113 test cases, in 52 (46.0%) the progesterone levels were found between 5 and 9 nmol/L and in these cases, the model could not predict the day. In the remaining 61 cases, the model was tested and a correct prediction was found in 56 cases (91.8%).

## Discussion

Knowing the day of ovulation is important for both women and the medical team. Correct ovulation timing is highly significant for women who wish to conceive naturally or women undergoing intrauterine insemination or the transfer of frozen embryos during a natural cycle.

We investigated the hormonal alterations associated with ovulation to better identify ovulation time and to give further uniform and precise tools for researchers and doctors.

Defining what is ovulation can be challenging since the definitions might have different applicabilities. Surprisingly, there is no yet clear consensus definition of this major event. Many studies rely on the disappearance of the leading follicle^16–18^ since it is the most consistent direct sign and as such, a highly reliable characteristics of ovulation. A study on 271 ovulation cycles showed 84.3% sensitivity and 89.2%, specificity for follicular rupture to ovulation (19). Therefore, we focused on hormonal changes regarding follicle rupture. The question of how to calculate the time of frozen embryo transfer, intrauterine insemination, or intercourse, whether it is based on follicular rupture, LH surge, or progesterone levels, is beyond the scope of this article.

In this study, LH, estrogen, and progesterone—three hormones that are crucial to the ovulation process were examined. The LH surge has been studied extensively over the years^19,20^. However, there is still no consensus in the literature on defining the LH surge^5,18^. Early study^21^ collected data from 21 normally cycling women, finding plasma LH levels at peak to be 3.8 times greater than follicular phase levels and reaching a mean LH peak plasma levels of 76.3 IU/L. In another study, it was suggested that LH surge is considered to occur if the LH levels increased by at least 180% from the 24 h previous measured value^22^ while others used an absolute cutoff of serum LH level of 10 IU/L^23^, 15 IU/L^24^, 17 IU/L^25^ or 20 IU/L or more^26^ to indicate the LH surge. Interestingly, the correlation between these LH surge definitions and their ability to predict ovulation has not been examined. As illustrated in this work, relative changes in LH levels did not produce satisfactory predictive values in both a 100% or 200% increase (low specificity for ≥ 100% cut off of 52.4% or low sensitivity of 39.7% for the ≥ 200% cutoff). Testing absolute values of LH as ovulation predictors was also found to have limitations. A cutoff LH level threshold of 40 IU yielded the best results, with a predictive value of 91%. Yet in this case the rate of FN was 28%. Hence our conclusion is that, in contrast to previous studies, relying only on the absolute value of LH level or comparing relative increase, results in relatively high FP and FN rates. Therefore, in the clinical setup, we recommend combining the LH surge determination with other hormone levels.

The changes in estrogen levels during the menstrual cycle have been widely studied throughout the menstrual cycle. Estrogen levels rise steadily during the follicular phase, peak just before ovulation, and begin to decrease afterward. Any estrogen decrease is 100% indicative that the patient being examined has already ovulated (D0 and after) or is going to ovulate the next day D(− 1). If ultrasound is performed concomitantly, it can easily be defined which of these two possibilities is true. Several studies^25,27^ have shown the significance of estrogen drops as an ovulation predictor. When ovulation was anticipated at the 17 IU/L cutoff for LH levels in conjunction with a 30% estradiol level decline, Irani et al. demonstrated superior frozen embryo transfer outcomes. Additionally, our results demonstrate that estrogen falls toward ovulation occurs gradually throughout the two days before ovulation as opposed to at a constant rate (average 21.3% reduction from D(− 2) to D(− 1) and 58 2% drop from D(− 1) to D(0). Hoff and his peers^14^ also discussed the reduction in estrogen in five cases. His data exhibits a similar two-phase estrogen drop upon careful analysis**.** Initially moderate decline lasting around 24 h from the start of the LH surge, followed by a rapid decline up until ovulation. In conclusion, the variations in estrogen levels around ovulation are crucial for pinpointing the exact time of ovulation because any fall in estrogen levels indicates that ovulation is about to occur. It is crucial to stress that when a reduction is seen, ovulation will or has already occurred can be predicted. However, as we have shown in 19% of cases, the absence of a decrease does not preclude ovulation the following day. LH and progesterone levels should therefore be taken into account if no decline is seen**.**

We further examined whether preovulatory progesterone absolute levels can predict efficiently ovulation time. It is known that progesterone levels are low throughout the follicular phase and begin to increase towards ovulation^14,28^. This progesterone surge was suggested by Dozortsev and his colleagues^29,30^ as the true physiological trigger of ovulation. In his study, Hoff claimed that progesterone increases 12 h before the onset of the LH surge and continues at a variable rate throughout and beyond the LH surge. Yet, in these works, the correlation between progesterone levels or rate of increase was not examined as a predictor of ovulation. We focused on whether pre-ovulatory progesterone absolute levels can predict efficiently ovulation time. The only finding that we were able to demonstrate was that progesterone level of ≥ 2 nmol/L before ovulation had high sensitivity but unfortunately a very low specificity. We conclude that preovulatory progesterone levels should not be used as a single predictor of ovulation but may have a role in combined hormone-based decisions.

The importance of progesterone in "ovulation" is related to its role in synchronizing the embryo and endometrium, and initiating the secretory transformation of the endometrium for implantation^20,31^. Our algorithm achieved better results when combining the follicular rupture and the different hormones than relying on a single hormone. We believe that not only progesterone, but other hormones such as the drop of estrogen are of import for the preparation of the endometrium for implantation.

We also investigated if the blood estrogen/progesterone ratio could predict the day of ovulation. Previous research looked into this question and concluded that a decrease in the ratio of estrogen/progesterone metabolites in urine corresponds to the day of luteal transition in ovulatory women^32,33^. Our research found no additional benefit to using blood estrogen/progesterone ratios over using each one separately. We hypothesize that if such a benefit exists, it is reflected in the models presented in this paper.

In terms of the post-ovulatory stage, it is known that patients who are scheduled for frozen embryo transfer, are occasionally evaluated when ovulation has already happened (no follicle on the ultrasound scan). We attempted to assess the predictive value of progesterone levels in predicting ovulation time retrospectively. Progesterone levels above 9 nmol/L had a 91.5% sensitivity for detecting D(+ 1) or later. The specificity, however, was low, at only 62.7%. If the patient arrives after ovulation, the low sensitivity makes it impossible to accurately detect the time of ovulation, and the embryo transfer should be canceled. However, we show that if the progesterone level is ≤ 5nmol/L and no follicles are observed, we can conclude that we are on ovulation day D(0), with a sensitivity of 55% and a specificity of 99.6%. Provided that during this cycle, follicle development was documented in the follicular phase, and endometrial thickness was sufficient, embryo transfer can be performed.

Since our findings have shown that there is some difficulty in predicting ovulation when examining each hormone individually, we decided to examine how integrating the accumulated information improves the clinician's ability to predict ovulation time. Figure 4 suggests a clinical algorithm for predicting the ovulatory day, based on all hormonal tests mentioned above. Our data-based algorithm provides an accuracy of 95–100% in determining ovulation time. We show that the integration of the different clinical parameters leads to better accuracy in determining ovulation time. Using these models, retrospective validation on NC-FET found a total of 97.9% accuracy in ovulation prediction.

We are aware of the limitations of this study. The model was developed using 118 cycles and validated using 113 cases. Further larger prospective studies are needed to confirm the model's conclusions.

In summary, the predictive values of the available hormone levels to assess ovulation are presented. An algorithm based on a combination of estrogen, progesterone LH, and ultrasound is presented, with good accuracy of 95–100% precision rate. These data can be helpful to both fertility physicians and general practitioners. More studies are needed to determine its effectiveness and impact on treatment outcomes compared to other predictive methods.

## Participants and methods

This prospective observational study was approved by the institutional review board of the Herzliya Medical Center and Informed consent was obtained from all participants. All participants were healthy nulliparous and normal-ovulatory volunteers. Participants did not consume any hormonal contraceptives or other medications including medications that could potentially impact hormone levels, including prolactin levels, hormonal function, or the hypothalamic-pituitary-ovarian axis. Exclusion criteria included a history of oligomenorrhea (menstrual cycle length of greater than 35 days), infertility, polycystic ovary syndrome (defined by Rotterdam criteria^34^), pelvic inflammatory disease, endometriosis, and obesity.

Participants were monitored by blood tests and vaginal ultrasound. Day one of the menstrual cycle was defined by the onset of menstrual bleeding. Transvaginal ultrasonography was performed to document endometrium thickness follicle growth and ovulation using the ultrasonic scanner Clearvue-350-ultrasound-system (Philips Medical Systems). Follicle size was calculated as the mean of two diameters. Follow-up started on days 8–10 of the menstrual cycle. The frequency of the tests was determined by the rate of the leading follicle growth every day or two days. When the leading follicle reached 16 mm, daily monitoring was performed and continued up to three days after ovulation occurred. The test was performed by the ultrasound technician team at the hospital, which during the study period included 4 operators. The operator was blinded to the results of the other tests. At the same time as the ultrasound test, levels of LH, estrogen, and progesterone were measured. The intra-assay and the inter-assay CV% was determined, using Roche Elecsys reagents. The intra-assay coefficient of variation (CV) was 4.2%, 0.8% and 2.8% for estrogen, LH and progesterone, respectively. While the inter-assay CV was 2.7%, 2.0% and 7.3% for estrogen, LH and progesterone, respectively.

The day of ovulation was determined retrospectively as the day of documented follicular rupture and considered as day 0 (D(0)) while acknowledging that the follicle disappearance occurred within the 24 h since the previous examination, The data presented in this work includes the data obtained from three days before ovulation (D(− 3)) through three days after the day of ovulation day (D(+ 3)). Blood levels of LH, estrogen, and progesterone were examined as potential indicators of ovulation. Only fully documented D(0) and D(− 1) were included and The prediction power of the various hormones was tested. In addition, a model was built utilizing the entire data set, and the ovulation time was computed using the combination of the evaluated characteristics.

After processing the data and developing the ovulation prediction model, a retrospective validation of the model was performed using natural ovulation follow-up cycles. For this purpose, natural cycle frozen-thawed embryo transfers performed in Herzliya Medical Center between September 2018 to February 2022 were included. Briefly, the protocol of NC-FET includes several follow-up visits consisting of blood tests (estrogen, progesterone, and LH) and ultrasound. Timing and frequency are determined by the attending physician. Determining the date of ovulation is used to schedule frozen embryo transfer according to embryo age at the time of freezing. Validation cycles included cycles from patients undergoing FET cycles, with fully documented D(0) and D(− 1) ultrasound and blood tests for mentioned hormones.

### Statistical analysis

After data collection, averages, standard error of means (SEM), sensitivity, specificity, positive and negative predictive values (PPV and NPV respectively), and accuracy were calculated for the hormone levels tested using the MED-CALC Diagnostic test evaluation calculator.

Box plot analysis was used to present the distribution by day of each hormone. Receiver operating characteristic (ROC) curve analyses were applied to evaluate the discriminative ability of each hormone and AUC (Area under the curve) with a 95% interval was calculated for each hormone. To test the predictability of relative changes in hormone levels, ROC analysis and AUC of relative changes in hormone levels between different days were performed. p < 0.05 was considered statistically significant. Analyses were carried out using SPSS 25.0.

The Coordinates of the ROC Curve tables were analyzed, and the optimal cutoffs were selected to keep high specificity and appropriate sensitivity. The selected cut-off values were used to construct an algorithm to determine the ovulation time.

## Data Availability

Data will be available upon the editor's request.
